# Motivation and Motor Control: Hemispheric Specialization for Approach Motivation Reverses with Handedness

**DOI:** 10.1371/journal.pone.0036036

**Published:** 2012-04-26

**Authors:** Geoffrey Brookshire, Daniel Casasanto

**Affiliations:** 1 Department of Psychology, The New School for Social Research, New York, New York, United States of America; 2 Neurobiology of Language Department, Max Planck Institute for Psycholinguistics, Nijmegen, The Netherlands; 3 Donders Institute for Brain, Cognition, & Behaviour, Radboud University, Nijmegen, The Netherlands; McMaster University, Canada

## Abstract

**Background:**

According to decades of research on affective motivation in the human brain, approach motivational states are supported primarily by the left hemisphere and avoidance states by the right hemisphere. The underlying cause of this specialization, however, has remained unknown. Here we conducted a first test of the *Sword and Shield Hypothesis* (SSH), according to which the hemispheric laterality of affective motivation depends on the laterality of motor control for the dominant hand (i.e., the “sword hand," used preferentially to perform approach actions) and the nondominant hand (i.e., the “shield hand," used preferentially to perform avoidance actions).

**Methodology/Principal Findings:**

To determine whether the laterality of approach motivation varies with handedness, we measured alpha-band power (an inverse index of neural activity) in right- and left-handers during resting-state electroencephalography and analyzed hemispheric alpha-power asymmetries as a function of the participants' trait approach motivational tendencies. Stronger approach motivation was associated with more left-hemisphere activity in right-handers, but with more right-hemisphere activity in left-handers.

**Conclusions:**

The hemispheric correlates of approach motivation reversed between right- and left-handers, consistent with the way they typically use their dominant and nondominant hands to perform approach and avoidance actions. In both right- and left-handers, approach motivation was lateralized to the same hemisphere that controls the dominant hand. This covariation between neural systems for action and emotion provides initial support for the SSH.

## Introduction

Emotional states are intimately linked to actions, and to the hands people use to perform them. *Approach* actions are usually performed with the dominant hand, and *avoidance* actions with the nondominant hand [1]–[2]. In centuries past, sword fighters wielded the sword in the dominant hand when *approaching* an enemy, and raised the shield with the nondominant hand to *avoid* attack [3].

This “sword and shield" pattern of hand use is easy to observe in more ordinary actions, as well [1]–[2]. The dominant “sword hand" is used preferentially to perform approach actions regardless of whether these actions are positive (e.g., picking up a piece of fruit that you want to eat) or negative (e.g., thrusting at an enemy with a sword). Likewise, the nondominant “shield hand" is used preferentially to perform avoidance actions regardless of whether these actions occur in response to something positive (e.g., shading your eyes from the Summer sun) or negative (e.g., raising your shield to fend off attack). As these examples illustrate, the dominant and nondominant hands tend to be used differentially for actions that differ in *motivation* – not necessarily for actions that differ in valence (motivation does not always co-vary with valence) [4]–[5], nor for actions involving flexion vs. extension movements (motivation does not always co-vary with flexion vs. extension) [6].

Here we investigated whether the sword and shield pattern of hand use is reflected in the hemispheric organization of affective motivation in the human brain. In right-handers, approach- and avoidance-related motivational states are differently lateralized in the cerebral hemispheres. According to dozens of studies, the left hemisphere is specialized for approach emotions, and the right hemisphere for avoidance emotions [5], [7]. This means that, for right-handers, approach motivation is co-lateralized with the neural circuits primarily responsible for control of the dominant hand, and avoidance motivation with circuits that control the nondominant hand. Casasanto [1] proposed that affective motivation and motor control may co-lateralize due to a functional relationship between motivational states and approach and avoidance hand actions, established either over evolutionary or developmental time. We call this the *Sword and Shield Hypothesis* (SSH). If the SSH is correct, then the hemispheric laterality of approach and avoidance motivation found previously in right-handers should reverse in left-handers, for whom cortical control of the “sword hand" (used for approach actions) and “shield hand" (used for avoidance actions) is reversed.

**Figure 1:**
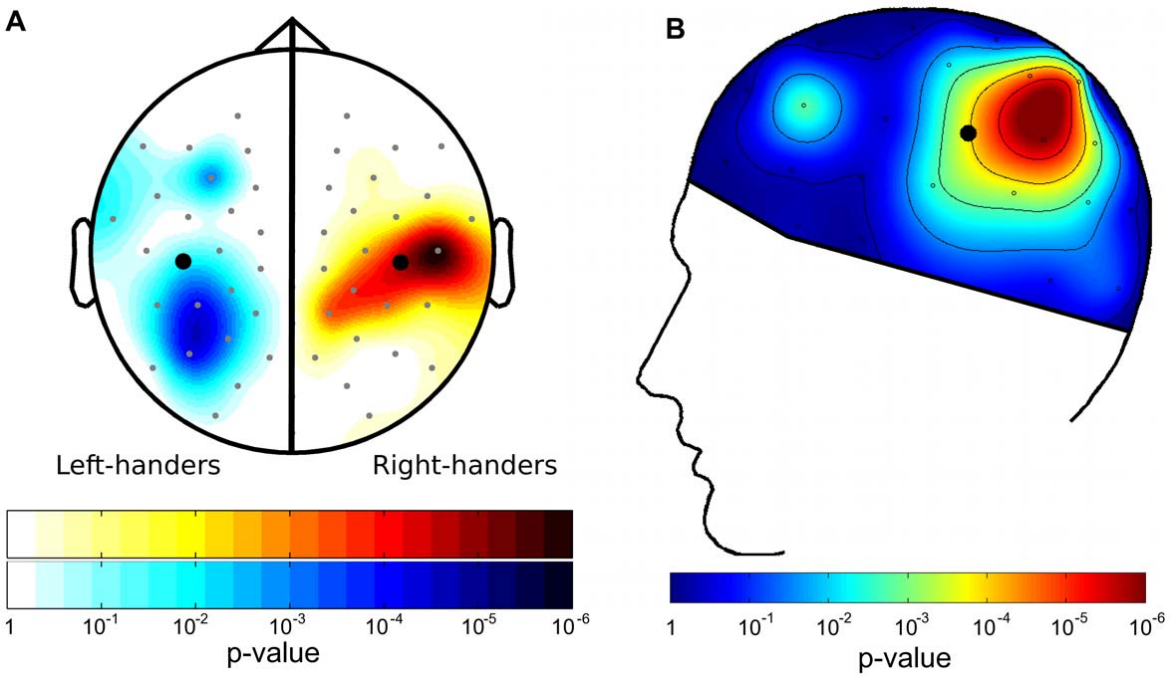
Hemispheric specialization for affective motivation depends on handedness. (**A**) Scalp topography of the statistical significance of the Approach Motivation×Hemisphere interaction on resting alpha-band power, computed and plotted separately in left-handers (left side) and right-handers (right side). Because this plot shows an interaction across hemispheres, each handedness group's topography is mirrored across the mid-sagittal line, and is therefore plotted on only one hemisphere. (**B**) Scalp topography of the statistical significance of the 3-way Approach Motivation×Hemisphere×Handedness interaction in right- and left-handers. This interaction is significant at 10 pairs of electrodes (*p*<.01). The highlighted electrodes were used for the analyses reported in the main text.

To test this prediction, we measured alpha-band (8–12 Hz) power in right- and left-handers during 3 minutes of resting-state electroencephalography (EEG), and analyzed hemispheric alpha-power asymmetries as a function of the participants' handedness and their approach motivational tendencies. Handedness was assessed with the Edinburgh Handedness Inventory (EHI) [8], and trait approach motivational tendencies were measured with the Behavioral Activation Scale (BAS) [9]. In right-handers, higher approach motivation has been shown to correlate with reduced alpha power (indicating increased neural activity [10]–[15]) during rest in the left hemisphere compared to the right hemisphere [16]–[18]. According to the SSH, the motivation-related alpha-power asymmetry typically found in right-handers should reverse in left-handers.

## Results

**Figure 2:**
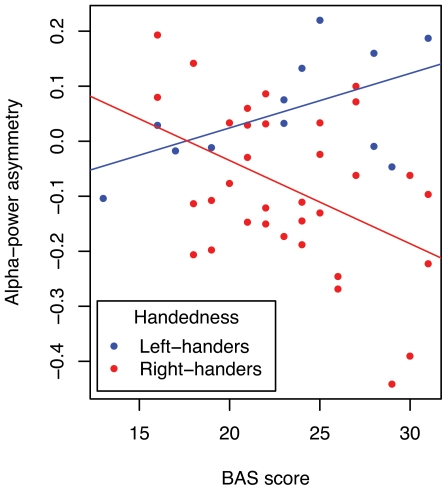
Associations between alpha power asymmetry and approach motivation in right- and left-handers. Asymmetries in *ln*-transformed alpha power are plotted for each subject as a function of BAS score. Asymmetry scores plotted here were computed as: (Left-hemisphere−Right-hemisphere)/(Left-hemisphere+Right-hemisphere). More positive values denote higher left hemisphere alpha power (and therefore less activity in the left hemisphere than in the right hemisphere). Alpha power for this plot was measured at the electrodes circled in **fig. 1**.

In right-handers, greater approach motivation was correlated with less alpha power (and therefore more neural activity) in the left hemisphere relative to the right hemisphere, indicating that their left hemisphere is specialized for approach motivation (BAS Score×Hemisphere interaction: Wald χ^2^(1) = 18.29, *p* = .00002; **fig. 1a** **, right side**). In left-handers, however, the opposite pattern was found, indicating that their *right* hemisphere is specialized for approach motivation (BAS Score×Hemisphere interaction: Wald χ^2^(1) = 6.08, *p* = .01; **fig. 1a** **, left side**). Combining data from right- and left-handers, Handedness (measured continuously using EHI Score) interacted with Motivation (BAS Score) and Hemisphere (Left, Right) to predict alpha power (Wald χ^2^(1) = 14.50, *p* = .0001; **fig. 1b** **;** **fig. 2**), confirming that the hemispheric correlates of motivation reversed with handedness. This 3-way interaction was found to be highly significant at 10 homologous electrode pairs across the scalp (all *p*<.01; **fig. 1b**). These included electrodes over superior temporal and parietal areas, as well as electrodes over a superior frontal site (near F3-4), where the alpha-power asymmetry has been observed most frequently in right-handers.

Prior studies of affective motivation and EEG alpha asymmetries have often been analyzed using Pearson correlations. This method of analysis collapses over epochs and obscures potentially informative variance in the data. To facilitate comparison with prior studies, however, we also report such a correlation analysis. Alpha asymmetry scores were calculated for each participant as ((Left-hemisphere−Right-hemisphere)/(Left-hemisphere+Right-hemisphere)) at the electrode pair highlighted in **fig. 1**. More negative asymmetry scores therefore indicate alpha power suppressions and greater neural activity in the left-hemisphere relative to the right. In right-handers, BAS scores correlated with alpha asymmetry (r = −.44, *p* = .009, **fig. 2**). In left-handers, the relationship between BAS and alpha asymmetry was marginally significant in the reversed direction (r = .56, *p* = .06), despite the small number of left-handers in our sample (N = 12). Crucially, the difference in these correlations was revealed to be significant by a z-test (z = 2.91, *p* = .004): This difference is analogous to the 3-way interaction of Handedness×Hemisphere×Motivation.

In contrast to other neuroimaging methods such as fMRI, there is not yet a widely accepted procedure for performing multiple comparisons corrections in EEG (but see [19]). We reanalyzed these data with Bonferroni corrections for each electrode pair, decreasing the critical p-value from 0.05 to (0.05/24) = 0.002. This analysis is overly conservative and may obscure real effects in the data. Still, the crucial three-way interaction remains significant at 7 electrode pairs, including the electrode pair highlighted in **fig. 1**.

## Discussion

Stronger approach-motivational tendencies were associated with more left-hemisphere activity in right-handers, but with more right-hemisphere activity in left-handers. Anatomical covariation between the neural substrates of affective motivation and of manual motor control is a prerequisite for the proposed functional relationship between action and emotion in the brain [1]. These results therefore provide initial support for the Sword and Shield Hypothesis: Approach motivation is differently lateralized in right- and left-handers' brains, consistent with (and perhaps because of) handedness-related differences in hemispheric specialization for manual motor control.

Cognitive and perceptual processes that interact strongly are often subserved by nearby cortical areas. Areas that subserve various aspects of language, for instance, are co-lateralized to the left hemisphere. Presumably, proximity facilitates information transfer among functionally related areas [20]. The co-lateralization of approach motivation with control of the dominant hand, therefore, is consistent with a functional connection between these neural circuits for emotion and action. Further experiments are needed to test for causal relationships between the neural substrates of motivation and motor control, and to determine whether these co-lateralized systems are also co-localized (i.e., overlapping) within the same hemisphere.

The SSH applies specifically to affective motivation (i.e., the drive to approach or withdraw from physical or social stimuli), and not to other components of emotion such as valence (i.e., the positivity or negativity of feelings or evaluations). In behavioral studies, left- and right-handers sometimes show opposite patterns of responses to stimuli with positive and negative valence. For example, right-handers typically rate faces to be more positive when they appear in the right visual hemifield (VHF), whereas left-handers may rate them to be more positive when they appear in the left VHF [21]–[22]. Some researchers have interpreted these findings as evidence that the hemispheric laterality of emotional valence reverses with handedness [21]–[22]. This conclusion has been called into question, however, on the basis of subsequent studies: Right- and left-handers tend to make opposite judgments about the positivity and negativity of stimuli presented on their right and left sides even when the stimuli are processed bi-hemispherically [1], [23]. Although motivation and valence have been conflated in the emotion literature for decades, there is now strong evidence that these basic components of emotion are dissociable [4], [5]. Therefore, experiments on valence may not be informative about the hemispheric laterality of motivation, or vice versa. The present study provides the first evidence that affective motivation is differently lateralized in right- and left-handers. It remains an open question whether emotional valence is also differently lateralized in the brain as a function of handedness.

Many cognitive functions show some degree of variation with handedness. Aspects of language and spatial cognition that are clearly lateralized in right-handers are more bilaterally distributed in left-handers [24]–[25]. The complete *reversal* of hemispheric specialization that we observe here, however, is rarely found – except in the motor system.

These findings have potential clinical implications. To decrease symptoms of depression, transcranial magnetic stimulation (TMS) is used to shift the balance of neural activity toward patients' left hemispheres, in order to stimulate approach-related emotions [26]. These lateralized neurostimulation therapies depend critically on the assumption that the left hemisphere mediates approach motivational states. Given the hemispheric reversal we show here, however, it appears that therapies that are beneficial to right-handers could be detrimental to left-handers.

On the basis of the alpha-power asymmetry in right-handers, the left-hemisphere locus of approach motivational states is widely accepted as a fact [5], just as the left-hemisphere locus of language (even in the majority of left-handers) is an established fact. The present findings, therefore, call for a substantial revision to the dominant model of the cortical organization of emotion [5], [27]–[29].

Furthermore, these results suggest that the hemispheric laterality of motivation is principled, not arbitrary, and may not pose an enduring mystery like the laterality of language has. Affective motivation co-lateralizes with manual motor control, consistent with the way people use their right and left hands differentially to perform approach and avoidance actions.

## Methods

### Ethics statement

All participants gave written informed consent before participating in this study, which was conducted in accordance with international standards for the ethical treatment of humans as experimental subjects and was specifically approved by the local ethics committee (Commissie Mensengebonden Onderzoek Region Arnhem-Nijmegen).

### Participants

Native Dutch speakers (N = 48, 13 males) participated in exchange for payment. Participants had no history of psychiatric disorders or brain injury. For consistency with prior studies, we excluded 2 participants who were not strongly handed (|EHI|≤25), leaving 34 right-handers (7 male; mean EHI = 83.1±17.0) and 12 left-handers (5 male; mean EHI = −80.5±13.8). Left-handers were recruited using a participant database that allowed us to screen for handedness. They were not aware that they were being recruited on the basis of their handedness.

### Procedure

Participants remained still during six 1-minute blocks of resting-state EEG. Each participant performed three blocks with their eyes closed and three with their eyes open, looking at a blank screen during the eyes-open blocks. Blocks alternated between open and closed eyes, with block order randomized between participants. After EEG, participants completed Dutch translations of the Behavioral Activation System (**Appendix S1**) [9] scale and the Edinburgh Handedness Inventory (**Appendix S2**) [8]. These scales were translated by a native speaker of Dutch and are included in the Supporting Information. There was no relationship between handedness and BAS (r = −0.004, *p*>.9), and BAS scores were indistinguishable between the handedness groups (Right-handers: 23.2±4.2; Left-handers: 23.0±5.7).

The Behavioral Inhibition System (BIS) scale is often administered along with the BAS scale [9]. However, several studies have failed to find an association between BIS and alpha-band activity in resting state EEG [17]–[18], [29]; but see [16], [19]. More importantly, the validity of BIS as a measurement of *avoidance motivation* has been called into question [17]–[18], [29], but see [16], [19]. Rather than avoidance motivation, BIS has been argued to index response inhibition [18], [29]. For these reasons, we did not test for any effects of BIS.

### EEG Recording

EEG was recorded with a 64-channel active electrode system, with the online reference electrode at the left mastoid and the ground at the nasion. Signals were sampled at 500 Hz with an online 1000 Hz low-pass filter and a 10 sec time constant (.016 Hz). Impedances between electrodes were reduced to 10 kΩ. Continuous EEG signals were segmented into 62-second epochs, including 1 sec at the beginning and end of each block of resting EEG.

### Data Analysis

Our analysis focused on only eyes-closed blocks, which provide the most sensitive measure of the relationship between alpha-power asymmetry and BAS [17]. On the basis of prior studies [16], [28], [30] and the scalp topography we observed in right-handers, one site was chosen for comparison across handedness groups (located approximately at T3-4). The statistical analyses reported in the main text were performed on alpha power recorded from this electrode pair (highlighted in **fig. 1**). This allowed unbiased selection of electrodes of interest for testing the left-handers and the relationship of hemisphere, BAS, and handedness. The significance of this critical 3-way interaction is illustrated for every homologous electrode pair in **fig. 1b**.

Signal processing and computation of time-frequency representations were performed using the FieldTrip package for Matlab [31]. Offline, all signals were mathematically re-referenced to the mean of the left and right mastoids, resampled to 300 Hz, and band-pass filtered between 2–30 Hz. Eye movement artifacts were excluded blind to the experimental condition with a semi-automated routine using principal component analysis. Time-frequency representations were computed in time steps of 50 ms, centered around 10 Hz with 2 Hz frequency smoothing and 500 ms time smoothing. Each alpha-power value, therefore, comprised the weighted average of activity from 8–12 Hz for an epoch from 250 ms before to 250 ms after the time point, convolved with a Hanning window. Activity for each 60 sec block was averaged and normalized by natural-log transformation. The *ln*-transformed average alpha-power of each block was analyzed using linear mixed-effects regressions with the *lmer* function in the *lme4* package in the R programming environment [32]. All p-values were computed with Wald χ^2^ tests comparing two models differing by one parameter at a time, using the *lme4::anova* function. Hemisphere (Left/Right), Handedness (measured continuously with EHI scores), and Approach Motivation (continuous with BAS scores) were entered into the models as fixed effects, and Subject was present in all models as a random effect.

For example, we first fit a model (**m1**) of right-handers' *ln*-transformed alpha power with a random effect for subject and fixed effects for Hemisphere, Motivation, and the Hemisphere×Motivation interaction. We then fit a second model (**m2**) with a random effect for subject and fixed effects for Hemisphere and Motivation, excluding the term for the 2-way interaction. A Wald χ^2^ test then determined if **m1** was a significantly better fit of the data than **m2**. If so, the Hemisphere×Motivation interaction was known to be statistically significant in predicting alpha power.

## Supporting Information

Appendix S1 **Dutch translation of the Edinburgh Handedness Inventory.** The EHI [8] was translated by a native speaker of Dutch.
(PDF)

Appendix S2 **Dutch translation of the Behavioral Activation System scale.** The BAS scale [9] was translated by a native speaker of Dutch.
(PDF)
