# Supplementary material for: Motivation and Motor Control: Hemispheric Specialization for Approach Motivation Reverses with Handedness
Source: PLoS One. 2012 Apr 26;7(4):e36036. doi: 10.1371/journal.pone.0036036 (PMC3338572; doi:10.1371/journal.pone.0036036)
Supplement: Appendix S1 — Dutch translation of the Edinburgh Handedness Inventory. The EHI [8] was translated by a native speaker of Dutch. (PDF) [file pone.0036036.s001.pdf]

Supplemental Information S1

Dutch translation of the Edinburgh Handedness Inventory

Geef hieronder met een vinkje (✓) aan welke hand je voorkeur heeft bij het uitvoeren van onderstaande taken.
Als je voorkeur zo sterk is dat je nooit je andere hand zou gebruiken, tenzij je daartoe gedwongen bent, zet dan twee vinkjes neer (✓✓).
Als je geen voorkeur hebt voor een bepaalde hand, zet dan een vinkje in allebei de colommen (✓ | ✓).
Voor sommige activiteiten gebruik je beide handen. In deze gevallen staat tussen haakjes aangegeven waarvoor precies je handvoorkeur wordt gevraagd.

Activiteit / Object	Linkerhand	Rechterhand
1. Schrijven		
2. Tekenen		
3. Gooien		
4. Schaar		
5. Tandenborstel		
6. Mes (zonder vork)		
7. Lepel		
8. Bezem (bovenste hand)		
9. Afstrijken lucifer (lucifer)		
10. Doos openmaken (deksel)		

Hieronder niet invullen

Total checks:	LH =	RH =
Cumulative Total	CT = LH + RH =	
Difference	D = RH – LH =	
Result	R = (D / CT) × 100 =	
Interpretation:<br>(Left Handed: R < -40)<br>(Ambidextrous: -40 ≤ R ≤ +40)<br>(Right Handed: R > +40)		

[1] Oldfield, R. C. (1971). The assessment and analysis of handedness: The Edinburgh inventory. *Neuropsychololgia*, *9*, 97-113.
