# Supplementary material for: Motivation and Motor Control: Hemispheric Specialization for Approach Motivation Reverses with Handedness
Source: PLoS One. 2012 Apr 26;7(4):e36036. doi: 10.1371/journal.pone.0036036 (PMC3338572; doi:10.1371/journal.pone.0036036)
Supplement: Appendix S2 — Dutch translation of the Behavioral Activation System scale. The BAS scale [9] was translated by a native speaker of Dutch. (PDF) [file pone.0036036.s002.pdf]

Supplemental Information S2

Dutch translation of the Behavioral Activation System scale items

When I get something I want, I feel excited and energized.

Als ik iets krijg wat ik graag wil, voel ik me energiek en enthousiast.

When I'm doing well at something, I love to keep at it.

Als ik goed ben in wat ik aan het doen ben, wil ik het graag blijven doen.

When good things happen to me, it affects me strongly.

Als me goede dingen overkomen, heeft dit een sterke invloed op me.

It would excite me to win a contest.

Het winnen van een wedstrijd maakt mij enthousiast.

When I see an opportunity for something I like, I get excited right away.

Als ik een kans om iets te doen wat ik leuk vind, ben ik meteen enthousiast.

When I want something, I usually go all-out to get it.

Als ik iets wil, dan ga ik er meestal helemaal voor om het te krijgen.

I go out of my way to get things I want.

Ik heb er veel voor over om te krijgen wat ik wil.

If I see a chance to get something I want, I move on it right away.

Als ik een kans zie om iets te krijgen wat ik wil, onderneem ik meteen actie.

When I go after something I use a "no holds barred" approach.

Als ik iets wil bereiken ben ik bereid hier hard voor te werken.

I will often do things for no other reason than that they might be fun.

Ik doe vaak dingen met geen andere reden dan dat het me leuk lijkt.

I crave excitement and new sensations.

Ik heb veel behoefte aan spanning en sensatie.

I'm always willing to try something new if I think it will be fun.

Ik ben altijd bereid om iets nieuws te proberen als ik denk dat het leuk is.

I often act on the spur of the moment.

Als ik dingen doe, laat ik me meeslepen door het moment.
